# Putative Mechanisms Underlying the Beneficial Effects of Polyphenols in Murine Models of Metabolic Disorders in Relation to Gut Microbiota

**DOI:** 10.3390/cimb44030091

**Published:** 2022-03-18

**Authors:** Yoshimi Niwano, Hidetsugu Kohzaki, Midori Shirato, Shunichi Shishido, Keisuke Nakamura

**Affiliations:** 1 Faculty of Nursing, Shumei University, Yachiyo 276-0003, Japan; kohzaki@mailg.shumei-u.ac.jp; 2 Department of Advanced Free Radical Science, Tohoku University Graduate School of Dentistry, Sendai 980-8575, Japan; midori.shirato.c8@tohoku.ac.jp (M.S.); shunichi.shishido.b2@tohoku.ac.jp (S.S.); keisuke.nakamura.e5@tohoku.ac.jp (K.N.)

**Keywords:** polyphenol, metabolic disorders, murine models, gut microbiota

## Abstract

The beneficial effects of polyphenols on metabolic disorders have been extensively reported. The interaction of these compounds with the gut microbiota has been the focus of recent studies. In this review, we explored the fundamental mechanisms underlying the beneficial effects of polyphenols in relation to the gut microbiota in murine models of metabolic disorders. We analyzed the effects of polyphenols on three murine models of metabolic disorders, namely, models of a high-fat diet (HFD)-induced metabolic disorder, dextran sulfate sodium (DSS)-induced colitis, and a metabolic disorder not associated with HFD or DSS. Regardless of the model, polyphenols ameliorated the effects of metabolic disorders by alleviating intestinal oxidative stress, improving inflammatory status, and improving intestinal barrier function, as well as by modulating gut microbiota, for example, by increasing the abundance of short-chain fatty acid-producing bacteria. Consequently, polyphenols reduce circulating lipopolysaccharide levels, thereby improving inflammatory status and alleviating oxidative imbalance at the lesion sites. In conclusion, polyphenols likely act by regulating intestinal functions, including the gut microbiota, and may be a safe and suitable therapeutic agent for various metabolic disorders.

## 1. Introduction

Polyphenols, widely distributed in fruits, vegetables, and plant-based beverages, such as tea, coffee, and wine, have health benefits, which have been thought to be due to their antioxidative activity. Polyphenols, alone or as part of mixtures, have been shown to prevent and alleviate oxidative stress-related metabolic disorders due to their intrinsic ability to scavenge free radicals by providing an electron or a hydrogen atom [1,2]. Although polyphenols have low oral bioavailability mainly because of their extensive biotransformation in the intestine and liver, as well as by the gut microbiota [3,4,5], they exert remarkable beneficial effects, which lead to the low bioavailability/high bioactivity paradox.

The human body provides an ecosystem for the habitation of trillions of microbial cells, most of which reside in the gastrointestinal tract; the gut microbiota is most likely associated with metabolic events related to health and disease [6]. The involvement of the gut microbiota in several pathophysiological conditions has been suggested [7], leveraging the advances in genomic techniques, such as 16S and 18S ribosomal RNA sequencing and metagenomic sequencing [8,9].

Besides their effects on oxidative stress-related metabolic disorders, polyphenols substantially interact with the gut microbiota [10,11]. Because an imbalance in the quantity and quality of gut bacteria is associated with several metabolic disorders, the interaction of polyphenols and gut microbiota has been focused upon [1], and in the past decade, several studies have been carried out in this regard. However, the fundamental mechanisms underlying the beneficial effects of polyphenols in relation to the gut microbiota have not yet been fully elucidated. Therefore, this review is aimed at describing the beneficial effects of polyphenols on metabolic disorders in light of their interactions with the gut microbiota.

As data on the effect of polyphenols on human gut microbiota based on human intervention studies are limited, we searched for studies on murine models of metabolic disorders in the PubMed database using the keywords “polyphenol AND gut microbiota AND (rat OR mouse)”. We selected articles from the last decade (2012–2021) discussing the relationship between the heath benefit action of polyphenols and the gut microbiota. Although polyphenols have a variety of compounds, such as flavonoids, phenolic acids, and lignans, we focused on polyphenols in general including plant extracts. Additionally, we excluded the literature discussing the prebiotic action and phytoestrogenic action of polyphenols.

## 2. Beneficial Effects of Polyphenols on Metabolic Disorders in Relation to the Gut Microbiota in High-Fat Diet (HFD)-Fed Murine Models

HFD-fed mice and rats have been used as in vivo models of chronic metabolic disorders, such as obesity, hyperlipidemia, hyperglycemia, liver injury, and inflammatory dysfunction, and the beneficial effects of polyphenols have been studied using these models in relation to the modulation of the gut microbiota [12,13,14,15,16,17,18,19,20,21,22,23,24,25,26,27,28,29,30,31,32,33,34] as summarized in Table 1. In particular, as indicated by a previous study [30], oxidative stress, inflammation, and gut microbial disorders can be induced by long-term HFD. Therefore, an HFD model seems to be suitable for evaluating the putative modes of the action of polyphenols. Of polyphenols, resveratrol, a well-known sirtuin 1 (SIRT1) agonist, has been intensively studied. Resveratrol, alone or in combination with other polyphenolic compounds such as quercetin and sinapic acid or probiotics such as *Bifidobacterium longum*, alleviates effects of obesity, hyperlipidemia, hyperglycemia, and nonalcoholic fatty liver disease (NAFLD) in HFD-fed mice [17,21,26,28,29,31]. Collectively, the beneficial effects of resveratrol are likely attributable to improved oxidative stress and gut microbial composition. Because the bioavailability of many polyphenols, including resveratrol, is low [35,36], they can be located in the bowel lumen when orally administered. Resveratrol can directly alter the composition of the gut microbiota by increasing the abundance of short-chain fatty acid (SCFA)-producing bacteria, such as *Bacteroides* and *Blautia*, and by decreasing the abundance of harmful bacteria, such as *Desulfovibrio* and *Lachnospiraceae_NK4A316_group*, as well as improving intestinal oxidative stress by preventing the production of reactive oxygen species (ROS) and improving antioxidant defense mechanisms, for example, by enhancing superoxide dismutase (SOD) and glutathione (GSH) levels [28,29]. The increase in these SCFA-producing bacteria could lead to the anti-obesity effects of resveratrol because these bacteria reportedly correlate negatively with inflammation, insulin resistance, and obesity [37,38]. Indeed, fecal microbiota transplantation (FMT) from resveratrol-treated mice to HFD-fed mice resulted in decreased weight gain and increased insulin sensitivity in the latter [26]. Furthermore, resveratrol could improve the integrity of the gut intestinal barrier through the repair of intestinal mucosal morphology possibly due to improved intestinal redox status, which leads to amelioration of HFD-induced NAFLD [28], because the development of HFD-induced NAFLD is closely associated with a loss of tight junction proteins in the small intestine [39,40].

Aside from resveratrol, other polyphenols and polyphenol-rich extracts and substances also alleviate obesity, hyperlipidemia, liver injury, and inflammatory status secondary to the alteration of the gut microbiota composition [12,13,14,15,16,18,19,20,22,23,24,25,27,30,32,33,34]. Sinapine, a rapeseed polyphenol, ameliorated NAFLD, suppressed intestinal nuclear factor-κB (NF-κB) and tumor necrosis factor-α (TNF-α) expression, and enhanced adipose tissue insulin receptor substrate 1 (IRS-1) expression in HFD-fed mice [22]. Sinapine possibly manifested its effect by modulating the composition of the gut microbiota by decreasing the ratio of Firmicutes to Bacteroidetes and increasing the abundance of probiotics. Phylum-level analyses of Firmicutes and Bacteroidetes have revealed that a reduced population of Bacteroidetes or an increased population of Firmicutes is associated with obesity [41,42,43,44,45]. Among plant-origin polyphenol-rich extracts, the extracts of pomegranate peel, cranberry, cinnamon bark, grape and grape pomace, brown macroalga *Lessonia trabeculata*, *Lonicera caerulea* L. berries, and red pepper could attenuate obesity, hyperglycemia, or liver injury by alleviating oxidative stress and modulating the gut microbiota [12,14,15,19,24,25,27,32]. Polyphenol-rich beverages, food, and their ingredients also show beneficial effects on metabolic disorders in relation to the gut microbiota [13,16,18,23,30,33,34]. Tea, a popular beverage consumed worldwide, is known to contain catechins, such as epicatechin, epicatechin-3-gallate, epigallocatechin, and epigallocatechin-3-gallate (EGCG) [46]. Oral administration of tea polyphenols, such as polyphenols from fermented Pu-erh tea and fermented Fu brick tea mainly produced in China, ameliorated obesity and hyperlipidemia by ameliorating effects of inflammation and oxidative stress in the intestine, and improved intestinal barrier function, leading to reduced circulation of lipopolysaccharides (LPS) and modulation of the gut microbiota [16,18,23,34]. The tea polyphenols decreased the abundance of Proteobacteria, a source of LPS, and the Fu brick tea polyphenols increased phylogenetic diversity and decreased the Firmicutes/Bacteroidetes ratio. Pu-erh tea polyphenols reduced circulation of LPS via restoration of gut barrier function and restored HFD-induced gut microbial community structural shift. Chronic consumption of commercially available instant caffeinated coffee also ameliorated obesity and decreased the Firmicutes/Bacteroides ratio [13]. Polyphenol extracts from Shanxi-aged vinegar showed similar effects [30]. Polyphenol-rich whole red grape juice could reduce oxidative stress and inflammatory status by activating the body’s antioxidant system, preventing free radical action, and beneficially modulating the gut microbiota, although it slightly affected the body composition such as body mass, fat mass, and lean mass, and body bone area [33].

**Figure 1:**
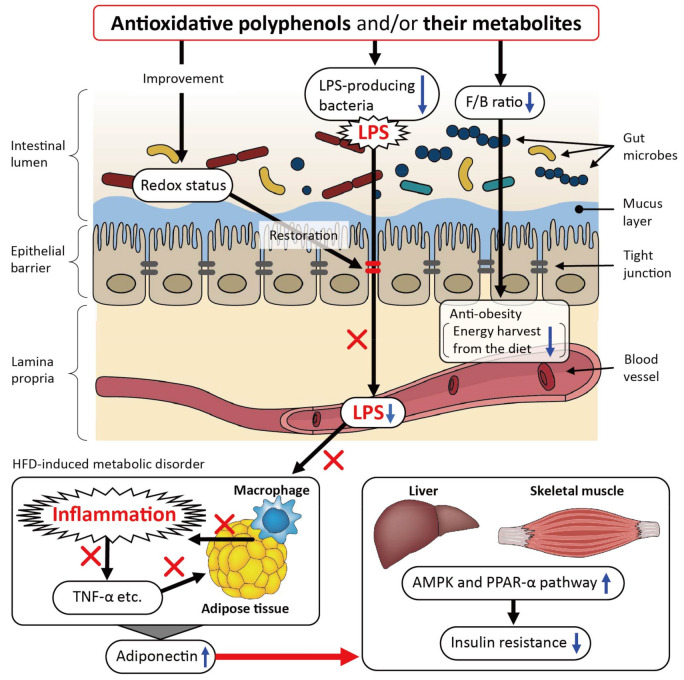
The proposed mechanism underlying the beneficial effects of polyphenols on metabolic disorders in high-fat diet (HFD)-fed murine models. LPS: lipopolysaccharide; ROS: reactive oxygen species; F/B ratio: Firmicutes/Bacteroidetes ratio; AMPK: AMP-activated protein kinase; PPAR-α: peroxisome proliferator-activated receptor-α.

The proposed mode of action of polyphenols in HFD-induced metabolic disorders is illustrated in Figure 1. As reported previously, metabolic endotoxemia dysregulates the inflammatory tone mediated by infiltrated macrophages and triggers body weight gain and diabetes [47], and a previous meta-analysis revealed that elevated levels of inflammatory cytokines (interleukin (IL)-1β, IL-6, IL-18, C-reactive protein) and TNF-α and low levels of adiponectin, an antidiabetic and antiatherogenic adipokine, are risk factors for type 2 diabetes [48]. Polyphenols decrease the abundance of bacteria, which are a source of LPS, and restore impaired intestinal tight junctions, possibly by improving the intestinal redox status, leading to lowered circulating LPS levels and alleviated inflammation in the adipose tissue. Subsequently, the levels of circulating adiponectin, an antidiabetic and antiatherogenic adipokine, can be restored, resulting in mitigating insulin resistance. This effect of adiponectin on insulin resistance appears to be mediated, at least in part, by an increase in fatty acid oxidation through activation of AMP-activated protein kinase (AMPK) and also through the peroxisome proliferator-activated receptor (PPAR)-α in the muscles and liver [49,50,51,52,53]. The polyphenols also decrease the Firmicutes/Bacteroidetes ratio, leading to an anti-obesity effect by depressing the increased capacity for energy harvest from the diet associated with obesity [44].

## 3. Beneficial Effects of Polyphenols on Dextran Sulfate Sodium (DSS)-Induced Colitis in Relation to the Gut Microbiota in Murine Models

**Figure 2:**
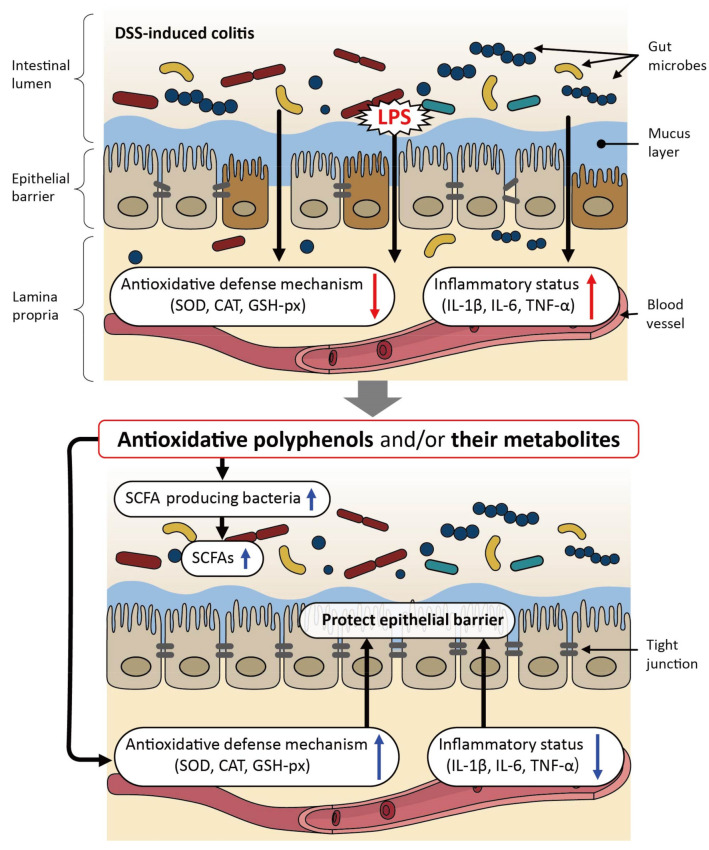
The proposed mechanism underlying the beneficial effects of polyphenols on dextran sulfate sodium (DSS)-induced colitis in murine models. SOD: superoxide dismutase; CAT: catalase; GSH-px: glutathione peroxidase; IL: interleukin; TNF: tumor necrosis factor; SCF: short-chain fatty acid.

Inflammatory bowel disease (IBD), such as Crohn’s disease and ulcerative colitis, is a recurrent and multifaceted inflammatory disorder requiring long-term medication [54,55]. DSS-induced colitis is an animal model of IBD, which has been used to study the beneficial effects of polyphenols in relation to the modulation of the gut microbiota [56,57,58,59,60,61,62,63,64,65] as shown in Table 2. In these studies, the oral intake of single polyphenolic compounds, polyphenol-rich extracts, or polyphenol-rich food substances ameliorated DSS-induced colitis, enhanced colonic barrier integrity, improved oxidative balance and inflammatory status in the blood and/or colon, and modulated the gut microbiota. As for the prophylactic effects, oral pretreatment with bronze tomato extract, quercetin, quercetin monoglycosides, taxifolin, flavanonol, or EGCG prevented the development of DSS-induced colitis, suggesting that these polyphenols can prevent DSS-induced oxidative imbalance and changes in the microbial composition in the colon [58,60,63,64]. A study showed that rectal administration of EGCG tended to exacerbate DSS-induced colitis, indicating that the direct effects of this compound are unlikely to play a primary role in vivo [64]; we presume that biotransformed metabolite(s) of EGCG could be the main driver for its action. In contrast, FMT from EGCG-treated mice to DSS-treated mice (EGCG-FMT) resulted not only in the amelioration of colitis but also in an increased abundance of SCFA-producing bacteria, such as *Akkermansia*, which showed a positive correlation with antioxidative indices and a negative correlation with inflammatory indices [64]. These results suggest that gut microbiota modulation, especially an increase in SCFA-producing bacteria, and, subsequently, in functional SCFAs, plays a pivotal role in EGCG-treated mice with colitis. The proposed mode of action of polyphenols in DSS-induced colitis is illustrated in Figure 2. The DSS-induced intestinal and systemic oxidative imbalance can be ameliorated by polyphenols, leading to the restoration of the impaired epithelial barrier of the intestine. In addition, polyphenols can increase the number of SCFA-producing bacteria with a subsequent increase in SCFA production, further enhancing the epithelial barrier function.

## 4. Beneficial Effects of Polyphenols on Metabolic Disorders Not Associated with HFD or DSS in Relation to the Gut Microbiota in Murine Models

**Figure 3:**
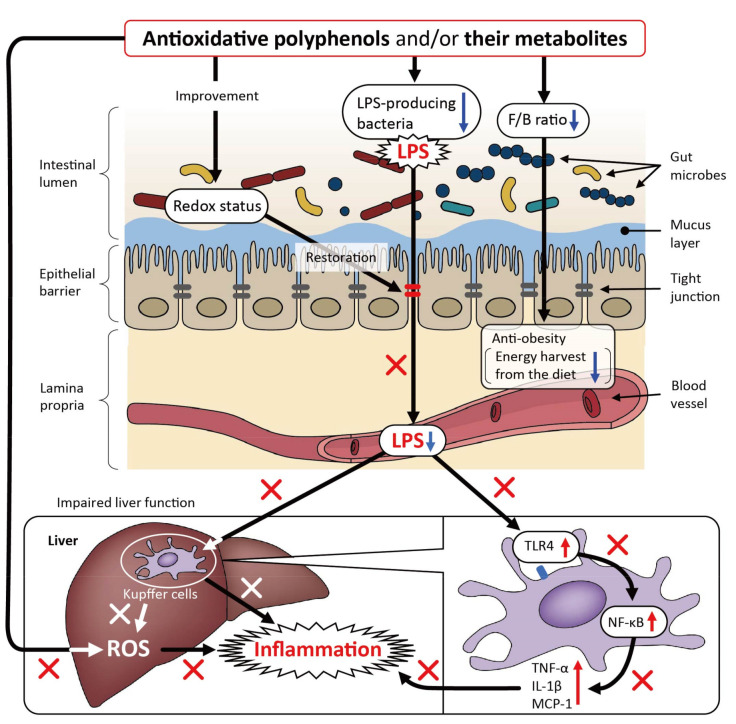
The proposed mechanism underlying the beneficial effects of polyphenols on murine liver injuries induced by various factors, except for a high-fat diet. LPS: lipopolysaccharide; ROS: reactive oxygen species; F/B ratio: Firmicutes/Bacteroidetes ratio; TLR: toll-like receptor; NF: nuclear factor; TNF: tumor necrosis factor; IL: interleukin; MCP: monocyte chemotactic protein.

The beneficial effects of polyphenols on metabolic disorders not associated with HFD or DSS are summarized in Table 3. Several studies have explored the beneficial effects on liver injury in relation to the gut microbiota. They showed that regardless of the hepatic disorder induced by different factors, such as fructose- or western diet-induced NAFLD, and alcohol-, LPS-, or L-carnitine-induced liver injury, polyphenols could prevent or alleviate liver injuries, ameliorate oxidative stress and inflammatory status, and modulate the composition of the gut microbiota or maintain its normal composition [66,67,68,69,70,71,72]. Polyphenol-treated animals show improved intestinal barrier function and reduced blood LPS levels, with the latter likely contributing to the prevention of necrotic damage to the liver. Four of the six studies on the taxonomic analysis of gut bacteria at the phylum level showed a clear decrease in the Firmicutes/Bacteroidetes ratio [66,67,68,71]. In studies on fructose- and ethanol-induced liver dysfunction [66,68], it was observed that the LPS content and Toll-like receptor 4 (TLR4) expression in the liver were decreased by oral intake of polyphenols. The latter study also showed that the abundance of Bacteroidetes was negatively correlated with parameters of oxidative stress and inflammation and that of Firmicutes was positively correlated; however, the role of the decreased Firmicutes/Bacteroidetes ratio in reduced liver inflammation was not discussed. Based on these results, a proposed mode of action of polyphenols on liver injuries induced by various factors is illustrated in Figure 3. Polyphenols can decrease the abundance of bacteria that are a source of LPS and enhance intestinal barrier function, possibly by improving intestinal redox status and lowering circulating LPS levels, which can, in turn, attenuate inflammation in the liver by suppressing the LPS-TLR4 signaling pathway in sinusoidal Kupffer cells.

Regarding metabolic disorders other than the liver injuries listed in Table 3, beneficial effects of polyphenols were reported on diabetic db/db mice, particulate matter ≤ 2.5 μm (PM_2.5_)-induced visceral adiposity, cafeteria diet-induced obesity, spontaneous hypertension, doxorubicin (an anti-cancer drug)-induced heart failure, and potassium oxonate-induced hyperuricemia [73,74,75,76,77,78]. Regardless of the experimental conditions and pathological sites, local and/or systemic oxidative stress-induced inflammation was reduced by polyphenol intake, along with altered gut microbiota. Regarding the involvement of gut microbiota in the actions of polyphenols, while in some studies it has been suggested that altered gut microbiota is the primary mechanism underlying the pharmacological actions of polyphenols [74,76], in others, it has been mentioned that further exploration is required to elucidate whether their beneficial effects are mediated by the gut microbiota [74,75,77].

## 5. Effects of Polyphenols on the Gut Microbiota in Healthy Mice and Rats

The effects of polyphenols on the gut microbiota of healthy animals have been reported [79,80,81,82] and are summarized in Table 4. A study showed that dietary supplementation of polyphenol-rich Jaboticaba (*Plinia jaboticaba*) peel extract altered the gut microbiota, increasing the abundance of *Lactobacillus*, *Bifidobacterium*, and *Enterobacteriaceae* without disturbing the antioxidant system [79]. *Lactobacillus* and *Bifidobacterium* were reported to exert inhibitory actions against harmful bacteria, likely via pH reduction [83]. Three other studies reported that polyphenol-rich dietary plant materials enhanced the hepatic antioxidant capacity and positively modulated the gut microbiota, even in healthy animals. The Firmicutes/Bacteroidetes ratio was significantly reduced by the dietary supplementation of whole golden kiwifruit with peel [80] and by oral gavage of polyphenol-rich *Penthorum chinense* extract [81]. Long-term oral gavage of anthocyanin-rich *Lycium ruthenicum* Murray was reported to increase SCFA-producing bacteria and enhance the intestinal barrier function [82]. These studies indicate that beneficial effects on the gut microbiota along with enhanced intestinal barrier function and/or antioxidant capacity could be exerted even in healthy animals.

## 6. Possible Involvement of Prooxidative Potential of Polyphenols in Intestinal Barrier Function

**Figure 4:**
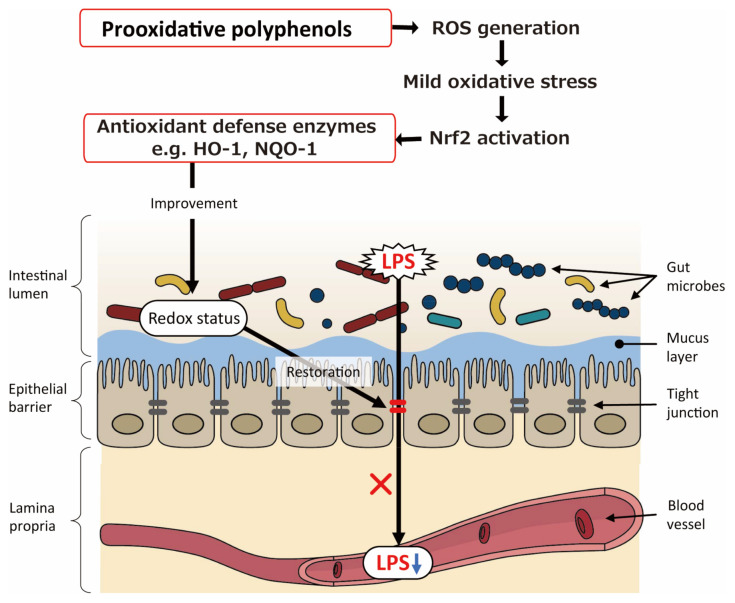
Schematic illustration of the possible involvement of prooxidative potential of polyphenols in intestinal barrier function. ROS: reactive oxygen species; Nrf2: nuclear factor E2-related factor 2; HO-1: heme oxygenase 1; NQO-1: NAD(P)H quinine oxidoreductase 1; LPS: lipopolysaccharide.

As described above, improved intestinal barrier function is likely a key player for polyphenols’ ameliorative action on metabolic disorders. It has been questioned if polyphenols directly exert antioxidative action in situ [84,85]. Aside from polyphenols’ direct antioxidative action, they possess prooxidative potential; e.g., antibacterial activity of catechins [86] and cytocidal action of plant polyphenols on cancer cells [87], both of which are exerted by cytotoxic ROS generated by oxidation of phenolic hydroxyl moiety coupled with the reduction of dissolved oxygen. Nuclear factor E2-related factor 2 (Nrf2) assumes the pivotal role in protecting cells, tissues, and organs owing to various genes encoding antioxidant proteins [88,89,90]. During ROS production by polyphenols, cells may activate the Nrf2 pathway independently of the polyphenols’ inherent antioxidant activity. This idea drove us to illustrate on possible involvement of prooxidative potential of polyphenols in intestinal barrier function (Figure 4). ROS generated by prooxidative polyphenols induces mild oxidative stress, which in turn activates Nrf2 followed by induction of antioxidant defense enzymes such as heme oxygenase 1 and NAD(P)H quinine oxidoreductase 1. These antioxidant enzymes could improve intestinal redox status, resulting in potentiated intestinal barrier function that prohibits LPS leakage to blood stream.

## 7. Future Perspective on Studies on Interaction of Polyphenols and Gut Microbiota

There are several issues that should be elucidated in the future studies. Given polyphenols’ poor absorbability from the digestive tract, their beneficial activity seems to be mediated through interaction with gut microbiota [91]. Accordingly, the number of studies on the interaction of polyphenols’ health beneficial effects and gut microbiota has gradually increased throughout this decade. Although many studies have shown that polyphenols could modulate gut microbiota, most studies failed to show how the polyphenols affected the microbiota on the basis of experimental evidence. In addition, while there have been many studies of polyphenol-rich plant extracts on this matter, there have been relatively a few studies of pure polyphenols. In other words, the possibility that components other than polyphenols could interact with gut microbiota still remains in the effects of plant extracts. Most of the studies performed chemical analyses of the polyphenols on the extracts; one study, for example, determined only the total polyphenol content along with carotenoid and capsinoid content, leaving us with the question of which component was a key player [32]. Next, despite the poor bioavailability of polyphenols, there have been very few reports on the in vivo fate of polyphenols in the literature we cited. To elucidate fundamental mechanisms, information on the absorption, distribution, metabolism, and elimination (ADME) of target polyphenols would be essential. Finally, future studies should also investigate whether polyphenol prooxidant properties are involved in improving intestinal barrier function along with the modulation of gut microbiota via the activation of Nrf2 pathway. Although meaningful findings have been accumulated through the efforts of many researchers, solving the above problem would give a new perspective to the in vivo effects of polyphenols in relation to gut microbiota.

Lastly, we also address the critical reviewing of the literature listed in the tables. Although replace, refine, reduce—the 3 Rs of ethical animal research—are globally accepted, researchers are required to formulate experiments based on enough statistical power to ensure the results of animal experiments, e.g., the message from UK funding agencies is that some experiments use too few animals, a problem that leads to wastage and low-quality results [92]. The National Institutes of Health also sounded a warning that some irreproducible reports using animal models are probably the result of coincidental findings that happen to reach statistical significance, coupled with publication bias [93]. In the tables, the number of animals per group in some papers were five or less, without stating the validity of the sample size [20,58,67,74]. We have to carefully interpret the data in such studies from the point of view of reproducibility.

## 8. Conclusions

There have been many reports on the beneficial effects of polyphenols on metabolic disorders, and recent studies have focused on their interaction with the gut microbiota. In HFD-fed murine models, polyphenols could ameliorate obesity, hyperlipidemia, and hyperglycemia by the alleviation of oxidative stress and inflammation in the intestine, the improvement of the intestinal barrier function, and the modulation of the gut microbiota, including a reduction in the Firmicutes/Bacteroidetes (F/B) ratio. In murine models of DSS-induced colitis, polyphenols could prevent or ameliorate oxidative imbalance, inflammatory status, and changes in the colonic microbial composition, with an increased abundance of SCFA-producing bacteria, leading to the protection of the intestinal epithelial barrier. In murine models of liver injuries not associated with HFD or DSS, polyphenols could improve the intestinal barrier function and reduced the blood LPS levels, which likely contributes to the prevention of necrotic damage in the liver, along with altered gut microbiota, including a reduction in the Firmicutes/Bacteroidetes ratio. Although some studies with FMT indicate a direct involvement of gut microbiota in the health benefits of polyphenols, further exploration is required in this regard.

Regarding the F/B ratio, it has been focused on by many researchers since the increased ratio was reported to be responsible for an increased capacity for energy harvest from diet [44]. Firmicutes and Bacteroides are the two main phyla of gut microbiota in mammals, playing important roles in maintaining gut microecological homeostasis [94]. Accordingly, it has been reported that alterations in the F/B ratio are associated with a variety of diseases [95,96,97]. However, there has been some inconsistency in the ratio even though similar experimental models were applied. For instance, in ovariectomized mice, one study showed an increased F/B ratio determined by a PCR analysis [98], but another one reported a decreased ratio determined by a 16s rDNA sequencing following DNA amplification by PCR [99]. In this review, five studies determined F/B ratios in Table 2, and among them one study revealed that the ratio increased [65], one study showed almost no change in the ratio [60], and the other three studies showed decreased ratios [56,58,63]. Thus, although the F/B ratio would be a good indicator to reflect gut microecological homeostasis, data should be carefully checked from the point of view of the following: which assay was applied for phylum level analysis, what timing of fecal sampling, how much the ratio changed, and so on.

Finally, the studies investigated thus far are limited to murine models, so that the findings cannot be extrapolated to humans. A review on the bioavailability of phytoestrogens such as isoflavones in murine models [100] noted that data should be carefully interpreted because of the large interspecies variability in the metabolism of phytoestrogens in murine models (e.g., their limited intestinal absorption and rapid excretion, compared to humans). Thus, data from murine models must be interpreted with great caution. In addition, given polyphenols’ poor absorbability in the digestive tract, their activity toward the human host seems to be mediated through interaction with intestinal microbes [101,102]. Considering transformation of dietary polyphenols by gut microbiota, reactions of polyphenols and bacteria are based on the reduction and/or hydrolysis because of anaerobic conditions. A typical well-known example is the bacterial transformation of the soya isoflavone daidzein to equal [103,104,105], which possesses high binding affinity to the estrogen receptor [106]. The *O*-deglycosylation of flavonoids by gut microbiota was also shown by many studies [102]. A typical example of flavonoid transformation is rutin to quercetin. As demonstrated, flavonoid aglycones, but not their glycosides, may inhibit growth of some intestinal bacteria [107], so that quercetin may have a more inhibitory influence on the intestinal bacteria than rutin. These direct effects of transformed polyphenols on gut microbiota have not been fully discussed in the literature listed in the tables. Since information on ADME of the polyphenols or the extracts is poor, further studies should be conducted in terms of ADME to obtain more information on the interaction between polyphenols and gut microbiota.

## Figures and Tables

**Table 1:** Beneficial effects of polyphenols in metabolic disorders in relation to the gut microbiota in high-fat diet (HFD)-fed murine models.

Reference, Publication Year, Animal Species, Polyphenol(s), and Dosage	Major Physiological Issues Improved	Mode of Action	
		Antioxidative and Anti-Inflammatory Action	Gut Microbiota Modulation
[12] 2013 Mice, polyphenol-rich pomegranate peel extract (PPE), p.o. in drinking water containing 0.2% PPE (average consumption of 6 mg/d per mouse) for 4 weeks	Reduced serum cholesterol (total and LDL) levels and alleviated tissue (colon and visceral adipose tissue) inflammation	−	Promoted the growth of gut bacteria, in particular, *Bifidobacterium* spp.
[13] 2014 Rats, p.o. as instant caffeinated coffee at a concentration of 20 g/L for 8 weeks (HFD was given for 10 weeks)	Reduced weight gain, adiposity, liver triglycerides, and energy intake	−	Decreased the Firmicutes/Bacteroidetes ratio
[14] 2015 Mice, high fat/high sucrose diet (HFHSD), polyphenol-rich cranberry extract, p.o. at 200 mg/kg/day for 8 weeks	Reduced visceral obesity and improved insulin sensitivity	Ameliorated oxidative stress and inflammation in the jejunum and reduced circulating LPS	Increased the relative abundance of *Akkermansia* spp.
[15] 2016 Mice, extractable polyphenol-rich fraction of table grapes (EP), p.o. with diet containing 1.1 g EP/kg for 16 weeks	Reduced white adipose tissue mass and improved glucose tolerance	−	Partially restored the HFD-mediated reduction in diversity
[16] 2016 Mice, green tea polyphenols (GTP), p.o. with a diet containing 0.05, 0.2, and 0.8% GTP for 8 weeks	Reduced obesity, and improved hepatic steatosis	−	Partially restored the HFD-mediated reduction in diversity
[17] 2017 Rats, a combination of quercetin (Q) and resveratrol (R), p.o. at 30 mg Q + 15 mg R/kg/day for 8 weeks	Reduced obesity	Attenuated serum inflammatory markers	Decreased the Firmicutes/Bacteroidetes ratio
[18] 2017 Mice, p.o. polyphenol- and caffeine-rich post-fermented Pu-erh tea, p.o. at 750 mg/kg/day for 12 weeks	Improved glucose and lipid metabolism disorder	Attenuated expression of inflammation genes in the proximal colon, reduced circulating LPS, and restored gut barrier integrity	Restored the HFD-induced gut microbial community structural shift
[19] 2018 Mice, polyphenol-rich cinnamon bark, or grape pomace extract (CBE or PBE), p.o. with a diet containing 0.2% CBE or and 0.8% PBE for 8 weeks	Reduced fat mass gain and adipose tissue inflammation, and ameliorated liver steatosis	Reduced adipose tissue inflammation, and improved gut barrier function	Decreased abundance of *Desulfovibrio* and *Lactococcus* at the genus level
[20] 2018 Mice, *Lonicera caerulea* L. berry polyphenols (LCBP), p.o. with diet containing 0.5% and 1% LCBP for 45 days	Improved hepatic steatosis	Attenuated serum inflammatory markers, and decreased LPS level in serum and liver	Decreased the Firmicutes/Bacteroidetes ratio
[21] 2019 Rats, resveratrol (RSV) and sinapic acid (SA), p.o. at 400 mg RSV/kg/day, 200 mg SA/kg/day, or a combination of RSV and SA for 8 weeks	Reduced fasting blood glucose levels and increased HDL-C levels by RSV	Decreased ROS and MDA levels in the colon, and increased total antioxidant capacity in the liver by SA	Combination of RSV and SA: Improved proportion of butyrate producer *Blautia* and *Dorea* from the *Lachaospiraceae* family and inhibited growth of bacterial species associated with diseases and inflammation, such as *Bacteroides* and *Desulfovibrionaceae* sp.
[22] 2019 Rats, sinapine (a rapeseed polyphenol), p.o. at 500 mg/kg/day for 12 weeks	Ameliorated NAFLD, reduced body weight and decreased TG and LDL-C levels.	Suppressed expression of NF-κB and TNF-α in the intestine and enhanced expression of IRS-1 in the adipose tissue	Decreased Firmicutes/Bacteroidetes ratio and increased abundance of probiotics, along with SCFA-mediated upregulation of G protein-coupled receptor 43 (GPR43) to inhibit the expression of inflammatory factors
[23] 2019 Mice, tea polyphenols (TPs) including EGCG, EGC, and ECG, p.o. at 100, 200, and 400 mg/kg/day for 12 weeks	Ameliorated hyperlipidemia, enhanced expression levels of hepatic lipid metabolism genes, and modulated gut microbiota	Maintenance of intestinal redox state by TPs	Decreased gut microbiota diversity and relative abundance of Proteobacteria, a source of LPS, possibly due to the antimicrobial activity of TPs
[24] 2019 Rats (treated with HFD + STZ), polyphenol-rich extracts from brown macroalga *Lessonia trabeculata* containing phlorotannin derivatives, phenolic acid derivatives, and gallocatechin derivatives, p.o. at 200 mg/kg/day for 4 weeks	Lowered fasting blood glucose and insulin levels, as well as better serum lipid profiles and antioxidant stress parameters	Increased response of antioxidant defense systems (e.g., CAT, SOD, and GSH in the liver) to oxidative stress	A positive effect on regulating the dysbiosis of the microbial ecology in diabetic rats
[25] 2019 Mice, pomegranate peel polyphenols including gallic acid, punicalagin, and catechin, p.o. at 150 and 300 mg/kg/day for 12 weeks	Alleviated obesity, decreased circulating proinflammatory cytokines, colonic tissue damage, and enhanced protein expression in the colonic tight junction	Improved oxidative damage and inflammation of the intestinal tissues, thereby reversing the reduced levels of tight junction proteins	Normalized the HFD-induced gut microbiota imbalance by increasing the abundance of beneficial bacteria in the colon
[26] 2020 Mice [fecal microbiota transplantation (FMT) to HFD-fed mice], resveratrol (RSV), p.o. at 300 mg/kg/day for 16 weeks followed by transplantation of the RSV-microbiota to HFD-fed mice (HFD-RSVT) to explore the function of the microbiota	HFD-RSVT decreased weight gain and increased insulin sensitivity	HFD-RSVT reduced the production of ROS and MDA in the intestine	A remarkable alteration in the composition of gut microbiota in mice treated with RSV, for example, enrichment of *Bacteroides*, *Lachnospiraceae_NK4A136_ group*, *Blautia*, *Lachnoclostridium*, *Parabacteroides*, and *Ruminiclostridium_9*, collectively referred to as RSV-microbiota
[27] 2020 Rats, *Lonicera caerulea* L. polyphenols containing anthocyanins, phenolic acids, and flavonoids, p.o. at 250 mg/kg/day for 8 weeks	Ameliorated intestinal permeability and intestinal inflammation; alleviated LPS-induced liver injury	Ameliorated intestinal oxidative stress damage (through regulation of the Nrf2/HO-1/NQO1 pathway)	Increased relative abundance of Bacteroidetes and Tenericutes and decreased relative abundance of Proteobacteria at the phylum level
[28] 2020 Mice (FMT from HFD-fed mice to HFD-fed mice), resveratrol (RSV), p.o. at 300 mg/kg/day for 16 weeks followed by transplantation of the HFDR-microbiota to HFD-fed mice (HFD-RSVT)	Alleviated NAFLD; ameliorated liver oxidative stress by HFD + RSV-microbiota treatment	HFD + RSV-microbiota treatment prevented HFD-induced production of ROS and improved antioxidant defense mechanisms (SOD and GSH levels)	The RSV-induced gut microbiota characterized by a decreased abundance of harmful bacteria, including *Desulfovibrio*, *Lachnospiraceae_NK4A316_group*, and *Alistipes*, as well as an increased abundance of SCFA-producing bacteria, such as *Allobaculum*, *Bacteroides*, and *Blautia*
[29] 2020 Mice, resveratrol (RSV), p.o. at 300 mg/kg/day for 16 weeks	Improved obesity	A two-part anti-obesity mechanism of RSV through the gut microbiota was proposed:(1) improved composition and function of the gut microbiota as well as the intestinal oxidative state; (2) 3-hydroxyphenylpropionic acid and 4-hydroxyphenylacetic acid (biotransformed from RSV by the gut microbiota), which may be responsible for the beneficial effects of RSV	
[30] 2020 Rats, polyphenol extracts from Shanxi-aged vinegar containing at least 41 polyphenols (including 18 phenolic acids), p.o. at 4, 8, and 16 mg/kg/day for 4 weeks	Improved hyperlipidemia	Improved inflammatory stress- and oxidative stress-related indicators	Decreased the Firmicutes/Bacteroidetes ratio; increased the diversity of microorganisms
[31] 2021 Mice, resveratrol (RSV) with probiotic *Bifidobacteria*, p.o. at 100 mg RSV/kg/day and probiotic *Bifidobacteria* for 3 weeks, starting the fifth week of HFD feeding	Coadministration of *B. longum* and RSV alleviated obesity and NAFLD	The combination of *B. longum* and RSV exerted an inhibitory effect on inflammatory cytokines and increased the levels of antioxidants, including SOD and GSH, and decreased the levels of MDA	RSV acted as an excellent prebiotic because most orally administered RSV is located in the bowel lumen
[32] 2021 Mice, *Capsicum annuum* L. ‘Senise’ extract (CAE) containing polyphenols, lycopene, and capsinoid derivatives, p.o. at 1, 10, and 25 mg/kg/day for 6 weeks	Promoted weight loss and improved plasma markers related to glucose and lipid metabolism	Reduced the expression of proinflammatory cytokines possibly due to the antioxidant property of CAE	Decreased the Firmicutes/Bacteroidetes ratio
[33] 2021 Rats, polyphenol-rich whole red grape juice, p.o. at 10 mL/day + physical training for 60 days	Lowered the concentration of IL-6 and TBARS	Reduced oxidative stress by activating the body’s antioxidant system, preventing the action of free radicals, and consequently, reducing the expression of inflammatory cytokines	The juice consumption beneficially modulated the gut microbiota
[34] 2021 Rats, Fu brick tea polyphenols, including EGCG, EGC, and ECG, p.o. at 100 mg/kg for 12 weeks	Improved the intestinal oxidative stress and intestinal barrier function, including intestinal inflammation and the integrity of the intestinal barrier		Attenuated HFD-induced gut microbiota dysbiosis, characterized by increased phylogenetic diversity and decreased Firmicutes/Bacteroidetes ratio

p.o., per os; ROS, reactive oxygen species; MDA, malondialdehyde; NAFLD, nonalcoholic fatty liver disease; NF-κB, nuclear factor-kappa B; TG, triglyceride; LDL-C, low-density lipoprotein cholesterol; IRS-1, insulin receptor substrate 1; EGCG, epigallocatechin gallate; EGC, epigallocatechin; ECG, epicatechin gallate; SCFA, short-chain fatty acid; LPS, lipopolysaccharide; STZ, streptozotocin; CAT, catalase; SOD, superoxide dismutase; GSH, glutathione; Nrf2, nuclear factor (erythroid-derived 2)-like 2; HO-1, heme oxygenase 1; NQO1, quinone oxidoreductase 1; FMT, fecal microbiota transplantation; IL-6, interleukin-6; TBARS, thiobarbituric acid-reactive substances; ―, not clearly described.

**Table 2:** Beneficial effects of polyphenols on dextran sulfate sodium (DSS)-induced colitis in relation to the gut microbiota in murine models.

Reference, Publication Year, Animal Species, Polyphenol(s), and Dosage	Major Physiological Issues Improved	Mode of Action	
		Antioxidative and Anti-Inflammatory Action	Gut Microbiota Modulation
[56] 2017 Mice, chlorogenic acid (CA), p.o. with drinking water containing 1 mM CA for 15 days; 2.5% DSS was given during the last 8 days with CA	Ameliorated DSS-induced colitis and improved mucosal damage	Suppressed the active NF-κB signaling pathway in the colon	Decreased the Firmicutes/Bacteroidetes ratio and increased the relative abundance of *Akkermansia*
[57] 2017 Mice, nanoparticle curcumin (NC), p.o. with diet containing 0.2% NC for 18 days; 3% DSS was given from day 8 to day 18	Ameliorated DSS-induced colitis and improved mucosal permeability	Suppressed NF-κB activation in colonic epithelial cells	Increased the abundance of butyrate-producing bacteria and fecal butyrate levels
[58] 2018 Mice, bronze tomato extract (BTE) rich in anthocyanins, flavonols, and stilbenoids, p.o. with diet containing 1% BTE for 2 weeks; 1% DSS was given from day 14 to day 29	Ameliorated DSS-induced colitis	Suppressed LPS-mediated production of pro-inflammatory cytokines	Decreased the Firmicutes/Bacteroidetes ratio
[59] 2018 Rats, polyphenol-rich Chinese propolis (CP) or Brazilian propolis (BP), p.o. at 300 mg/kg/day for 17 days; 3% DSS was given from day 7 for 1 week	Ameliorated DSS-induced colitis	Alleviated the intestinal oxidative status, and suppressed inflammatory gene expression in the distal colon	Reduced populations of *Bacteroides* spp.
[60] 2018 Mice, quercetin aglycone (Q) or quercetin aglycone with monoglycosides (Q + MQ), p.o. with diet containing 0.21% Q or 0.35% Q + MQ for 7 days; 3% DSS was given from day 8 for 1 week	Ameliorated DSS-induced colitis	Suppressed oxidative stress indicated by MPO, GSH, and MDA	Ameliorated reduced Firmicutes population and increased Proteobacteria population by DSS
[61] 2019 Mice, rape bee pollen extract rich in polyphenols including kaempferol, sinapic acid, and rosmarinic acid, p.o. at 10.6 and 21.2 g/kg/day for 15 days; 3% DSS was given from day 8 to day 12	Ameliorated DSS-induced colitis	Attenuated oxidative stress and downregulated the expression of inflammatory cytokines such as IL-1β	Reduced the abundance of *Allobaculum* and *Bacteroides*, and increased the abundance of *Lactobacillus*
[62] 2019 Rats, honey polyphenols including caffeic acid, chlorogenic acid, and rutin, p.o. at 10.5 mg/kg twice daily for 7 days; 3% DSS was given from day 0 to day 5	Improved DSS-induced colonic apoptosis and reduced the expression of inflammatory cytokines in the colon	Improved the levels of SOD, GSH-Px, NO, and MPO; downregulated the expression of IL-1β, IL-6, TNF-α, and IFN-γ genes, and upregulated the expression of IκB-α gene	Reduced the population of *Bacteroides*, *Corynebacterium*, and *Proteus* species at the genus level
[63] 2021 Mice, taxifolin, p.o. at 100 mg/kg/day for 7 days; from day 8, mice received 3% DSS for 7 days	Prevented DSS-induced colitis	Inhibited the secretion of proinflammatory cytokines, and increased the secretion of IL-10, secretory IgA, SOD, and immunoglobulins; increased the expression of intestinal tight junction proteins	Restored the microbiota composition in the colon, including the decrease in the abundance of Bacteroidetes and the Bacteroidetes/Firmicutes ratio at the phylum level
[64] 2021 Mice, Exp. I, epigallocatechin-3-gallate (EGCG), p.o. or rectal administration at 50 mg/kg/day for 3 days starting after supplementation with 2.5% DSS for 7 days; Exp. II, EGCG, p.o. at 50 mg/kg/day for 3 weeks with 2.5% DSS for the last 6 days; Exp. III, 2.5% DSS for 7 days followed by FMT for 3 days	Exp. I: oral, but not rectal, EGCG alleviated DSS-induced colitis; Exp. II: oral EGCG prevented DSS-induced colitis; Exp. III: FMT alleviated DSS-induced colitis	Oral, but not rectal, EGCG attenuated oxidative stress, and exerted an anti-inflammatory effect along with enhanced integrity of the colonic barrier; oral EGCG pre-supplementation induced beneficial outcomes similar to those achieved with oral EGCG administration	Attenuation of colitis by oral EGCG suggests an intimate involvement of SCFA-producing bacteria, of the genus *Akkermansia*.
[65] 2021 Mice, green pea (*Pisum sativum* L.) hull polyphenol extracts containing quercetin and its derivatives, kaempferol trihexanside, and catechin and its derivatives, p.o. at 100 and 600 mg/kg for 14 days; 3% DSS was given for 7 weeks from day 8	Alleviated DSS induced colitis	Restored oxidative balance, and regulated inflammatory factors along with repaired colonic function	Increased the Firmicutes/Bacteroidetes ratio, promoted the growth of *Lactobacillaceae* and *Lachnospiraceae*, and improved the level of SCFAs

p.o., per os; IL-1β, interleukin-1β; SOD, superoxide dismutase; GSH-Px, glutathione peroxidase; NO, nitric oxide; IL-6, interleukin-6; TNF-α, tumor necrosis factor-α; IFN-γ, interferon-γ; IκB-α, NF-κB inhibitor-α; IL-10, interleukin-10; IgA, immunoglobulin A; FMT, fecal microbiota transplantation; SCFA, short-chain fatty acids.

**Table 3:** Beneficial effects of polyphenols on metabolic disorders not associated with a high-fat diet (HFD) or dextran sulfate sodium (DSS) in relation to the gut microbiota in murine models.

Reference, Publication Year, Animal Model of the Disorder, Polyphenol(s), and Dosage	Major Physiological Issues Improved	Mode of Action	
		Antioxidative and Anti-Inflammatory Action	Gut Microbiota Modulation
[66] 2019 Mice with fructose-induced NAFLD, loquat fruit extract rich in polyphenols including chlorogenic acid, cryptochlorogenic acid, and oleanolic acid, p.o. at 25 and 50 mg/kg/day for 8 weeks	Prevented fructose-induced NAFLD with mitigation of abnormal body weight and improved lipid metabolism	Mitigated oxidative stress and inflammation; reduced the endotoxin content and improved fructose-induced breakage of the intestinal barrier	Maintained normal Firmicutes/Bacteroidetes ratio
[67] 2020 Mice with western diet-induced NAFLD, vine tea polyphenol extracted from *Ampelopsis grossedentata*, p.o. in drinking water (0.5, 1, and 2%) for 12 weeks	Decreased the serum levels of cholesterol and triglycerides, and reduced the accumulation of hepatic lipid droplets	Activated Nrf2-mediated expression of hemeoxygenase-1 and quinone oxidoreductase, and reduced hepatic TBARS levels to prevent hepatic oxidative stress	Increased the relative abundance of *Akkermansia*, and reduced the Firmicutes/Bacteroidetes ratio
[68] 2021 Mice with alcohol-induced liver inflammation, Zhenjiang aromatic vinegar (a traditional fermented food in China) rich in polyphenols including catechin, p-hydroxybenzoic acid, and vanillic acid, p.o. at 200 and 800 mg/kg/day for 30 days; EtOH was given p.o. after 32 h, every day	Protected against alcohol-induced liver injury	Inhibited oxidative stress (reduced the levels of ROS, iNOS, MDA, 4-HNE, and 8-OHdG) and LPS-mediated inflammation	Modulated the composition of the gut microbiota and improved gut immunity and intestinal homeostasis; decreased the Firmicutes/Bacteroidetes ratio
[69] 2021 Mice with alcoholic liver disease, ellagic acid, p.o. at 50 and 100 mg/kg/day for 4 weeks to mice that received 15% alcohol	Alleviated alcohol-induced liver injury	Alleviated hepatic antioxidant activities (GSH-Px, CAT, MDA, SOD, and GSH), and proinflammatory cytokines levels (IL-6, IL-1β, and TNF-α)	Improved the alcohol-induced gut microbiota dysbiosis; restored the relative abundance of microbiota, such as Firmicutes, Verrucomicrobia, Actinobacteria, Bacteroidetes, and Proteobacteria at the phylum level
[70] 2021 Rats with LPS-induced liver disease, *Aronia melanocarpa* polyphenols containing anthocyanins, flavonols, and hydroxycinnamates, p.o. at 50, 100, and 200 mg/kg/day with LPS (p.o. at 200 μg/kg/day) for 4 weeks	Alleviated the degree of LPS-induced liver disease	Alleviated LPS-induced oxidative stress in the liver (reduced ROS and increased GSH levels).	Modulated the composition of the gut microbiota and improved the intestinal barrier function. At the phylum level, the enrichment of Verrucomicrobia microflora was alleviated and the abundance of Actinobacteria was decreased
[71] 2021 Mice with L-carnitine-induced liver injury, chlorogenic acid, p.o. at 200 and 400 mg/kg/day for 12 weeks with 3% L-carnitine in drinking water	Prevented L-carnitine-induced liver injury	Inhibited free radical production and improved the antioxidant defense system; inhibited the inflammatory reaction (i.e., IL-1, IL-6, TNF-α, and TNF-β levels).	Inhibited the L-carnitine-induced increase in the abundance of Firmicutes and Proteobacteria, and promoted Bacteroidetes at the phylum level
[72] 2021 Mice with obesity and hepatic steatosis induced by a western diet (WD), low in fiber but high in fats and sugars, p.o. as the WD supplemented with 1% grape polyphenols rich in B-type proanthocyanidins	Higher lean mass and lower fat mass, body weight, and hepatic steatosis	Reduced the intestinal oxidative stress	Increased the abundance of *Akkermansia muciniphila*, a gut microbe reported to increase energy expenditure
[73] 2018 Diabetic db/db mice, polyphenol-rich extract of *Dendrobium loddigesi*, p.o. at 50 and 100 mg/kg/day for 8 weeks	Decreased blood glucose, LDL-C, and body weight	Inhibited oxidative stress (reduced MDA and increased SOD, CAT, and GSH) in liver and kidney, attenuated serum inflammatory markers (IL-6 and TNF-α)	Decreased the Firmicutes/Bacteroidetes ratio
[74] 2019 Mice exposed to PM_2.5_ by instillation, hydroxytyrosol, p.o. at 50 mg/kg/day for 4 weeks	Alleviated PM_2.5_-induced visceral adiposity and insulin resistance	Inhibited PM_2.5_-induced oxidative stress-mediated activation of NF-κB	Enrichment of gut microbiota, and reduction of pathogenic bacteria
[75] 2020 Rats fed an obesogenic cafeteria diet, hesperidin (a citrus polyphenol), p.o. at 40 and 100 mg/kg/day for 8 weeks	Decreased the total cholesterol, LDL-C, and free fatty acids; ameliorated blood pressure and insulin sensitivity, and decreased the markers of arterial stiffness and inflammation	Metabolomics revealed an improvement in lower excretion of inflammation- and oxidative stress-related metabolites	Excretion of higher amounts of microbe-derived metabolites, which positively correlated with the *Bacteroidaceae* family
[76] 2021 Spontaneously hypertensive rats, *Litchi chinensis* seed extract, rich in polyphenols, including procyanidins, cinnamtannins, and rutin, p.o. at 30 and 60 mg/kg/day for 10 weeks	Reduced blood pressure and alleviated hypertension-induced renal damage	Attenuated oxidative stress and inflammation	Increased the relative abundance of *Lactobacillus* and production of SCFAs in the intestine
[77] 2021 Mice with doxorubicin (DOX)-induced heart failure, purified polyphenols from *Arctium lappa* L. including arctiin, dicaffeoyl succinoylquinic acid, and luteolin, p.o. at 50 and 150 mg/kg/day for 29 days; on day 22, 24, and 26, DOX was i.p. injected	Reduced heart failure syndrome and reduced serum activities of casein kinase and lactate dehydrogenase	Alleviated serum oxidative stress and reduced serum levels of inflammatory indices (TNF-α and NO).	Increased the abundance of *Lactobacillaceae*, *Muribaculaceae*, and *Ruminococcaceae* and decreased the abundance of *Proteobacteria*, *Enterobacteria*, and *Escherichia_Shigella*; enhanced the abundance of bacteria producing SCFAs
[78] 2021 Mice with potassium oxonate-induced hyperuricemia, *Camellia japonica* bee pollen extract containing polyphenols including kaempferol, quercetin, and gallic acid, p.o. at 2 and 4 g/kg/day for 3 weeks; starting on the 15th day, mice received potassium oxonate for the following 7 days	Reduced serum uric acid by inhibiting XOD activity and improved renal function	Increased antioxidant biomarkers, SOD activity, and GSH content, and decreased MDA content in the liver	Increased the abundance of *Lactobacillus* that has an anti-hyperuricemia effect; decreased the Firmicutes/Bacteroidetes ratio

p.o., per os; NAFLD, nonalcoholic fatty liver disease; PM_2.5_, particulate matter (≤2.5 μm); NF-κB, nuclear factor-κB; LDL-C, low-density lipoprotein-cholesterol; Nrf2, nuclear factor (erythroid-derived 2)-like 2; TBARS, thiobarbituric acid reactive substances; ROS, reactive oxygen species; iNOS, inducible nitric oxide synthase; MDA, malondialdehyde; 4-HNE, 4-hydroxy-2-nonenal; 8-OHdG, 8-hydroxy-2′-deoxyguanosine; LPS, lipopolysaccharide; GSH, glutathione; TNF-α, tumor necrosis factor-α; NO, nitric oxide; SCFAs, short-chain fatty acids; XOD, xanthine oxidase; SOD, superoxide dismutase; IL-6, interleukin-6; IL-1β, interleukin-1β; TNF-β, tumor necrosis factor-β.

**Table 4:** Effects of polyphenols on the gut microbiota in healthy mice and rats.

Reference, Publication Year, Animal Species, Polyphenol(s), and Dosage	Observations	
	Antioxidative and Anti-Inflammatory Action	Gut Microbiota Modulation
[79] 2019 Rats, Jaboticaba (*Plinia jaboticaba*) peel extract containing gallic acid, flavonols, and anthocyanins, p.o. in drinking water (5 g/L) for 15 and 49 days	No disturbance of the antioxidant system	Increased the counts of *Lactobacillus*, *Bifidobacterium*, and *Enterobacteriaceae*
[80] 2020 Rats, freeze-dried whole golden kiwifruit (*Actinidia chinensis*) containing polyphenols, including quercetin, catechin, and chlorogenic acid, p.o. 4.6 g/kg/day for 28 days	Effectively improved the hepatic lipid profile, lipid peroxidation, long-chain fatty acid profile, and intestinal homeostasis	Reduced the Firmicutes/Bacteroidetes ratio at the phylum level; increased the abundance of beneficial bacteria (*Lactobacillus* and *Barnesiella*) and reduced the abundance of harmful bacteria (*Enterococcus*, *Escherichia*, and *Staphylococcus*)
[81] 2020 Mice, *Penthorum chinense* extract containing polyphenols, including pinocembrin-7-*O*-glucoside, thonningianin A, and brevifolin carboxylic acid, p.o. at 0.4 g/kg/day for 5 days	Increased antioxidant capacity (increased plasma SOD and hepatic CAT and GSH-px levels)	Increased microbiota diversity, elevated the Bacteroidetes/Firmicutes ratio at the phylum level, and enriched gut health-promoting bacteria
[82] 2020 Mice, anthocyanin-rich *Lycium ruthenicum* Murray (ACN), p.o. at 200 mg/kg/day for 12 weeks	Increased the antioxidant status in the liver (increased AOC, SOD, CAT, GSH, and GSH-Px levels and decreased the MDA levels); ameliorated the anti-inflammatory status in the colon (reduced the expression of iNOS, COX-2, TNF-α, IL-6, IL-1β, and IFN-γ mRNAs) and increased the intestinal barrier function (increased the expression of ZO-1, Occludin, Claudin-1, and Muc1 mRNAs)	Increased the proliferation of *Barnesiella*, *Alistipes*, *Eisenbergiella*, *Coprobacter*, and *Odoribacter*; increased SCFAs in the cecal content and feces.

p.o., per os; SOD, superoxide dismutase; CAT, catalase; GSH-Px, T-AOC, total antioxidant capacity; GSH, glutathione; MDA, malondialdehyde; iNOS, inducible nitric oxide synthase; COX-2, cyclooxygenase-2; TNF-α, tumor necrosis factor-α; IL-6, interleukin-6; IL-1β, interleukin-1β; IFN-γ, interferon-γ; ZO-1, zonula occludens-1; Muc1, mucin 1; SCFAs, short-chain fatty acids.

## Data Availability

Not applicable.
